# Nucleic Acid Aptamers: An Emerging Tool for Biotechnology and Biomedical Sensing

**DOI:** 10.3390/s150716281

**Published:** 2015-07-06

**Authors:** Ti-Hsuan Ku, Tiantian Zhang, Hua Luo, Tony M. Yen, Ping-Wei Chen, Yuanyuan Han, Yu-Hwa Lo

**Affiliations:** 1 Department of Electrical and Computer Engineering, University of California San Diego, La Jolla, CA 92093-0407, USA; E-Mails: tiku@eng.ucsd.edu (T.-H.K.); luohuabox@gmail.com (H.L.); yuh054@ucsd.edu (Y.H.); 2 Materials Science and Engineering Program, University of California San Diego, La Jolla, CA 92093-0418, USA; E-Mail: tiz022@eng.ucsd.edu; 3 College of Basic Medicine and Forensic Medicine, Sichuan University, Chengdu 610041, China; 4 College of Manufacturing Science and Engineering, Sichuan University, Chengdu 610041, China; 5 Department of Bioengineering, University of California San Diego, La Jolla, CA 92093-0412, USA; E-Mail: tmyen@eng.ucsd.edu; 6 Chemical Engineering Program, University of California San Diego, La Jolla, CA 92093-0448, USA; E-Mail: pingwei320@gmail.com

**Keywords:** DNA, RNA, aptamer, SELEX, biomarkers, polymer, liposome

## Abstract

Detection of small molecules or proteins of living cells provides an exceptional opportunity to study genetic variations and functions, cellular behaviors, and various diseases including cancer and microbial infections. Our aim in this review is to give an overview of selected research activities related to nucleic acid-based aptamer techniques that have been reported in the past two decades. Limitations of aptamers and possible approaches to overcome these limitations are also discussed.

## 1. Introduction

**Figure 1:**
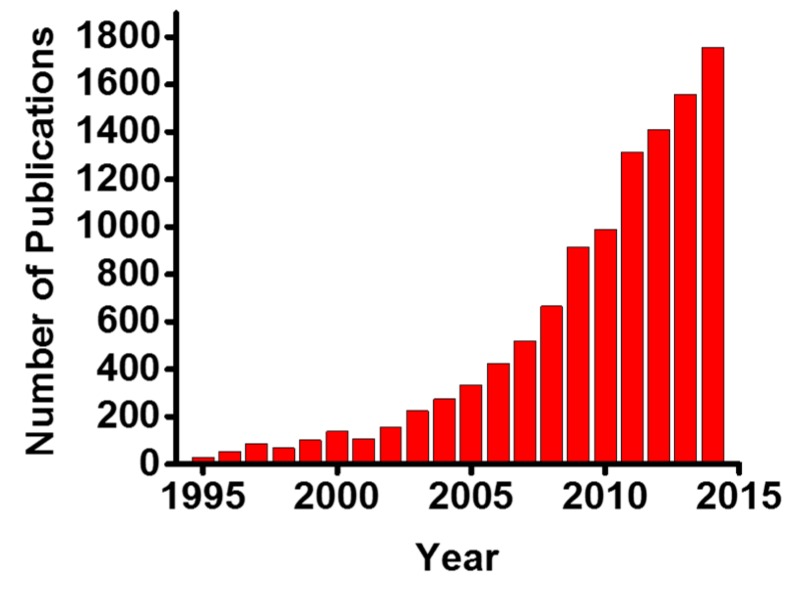
The annual trends in the number of publications of aptamers-related research. The term “aptamer” has been utilized for literature search on Thomson Reuters Web of Science.

Aptamers are short single-stranded DNA- or RNA-based oligonucleotides that can selectively bind to small molecular ligands or protein targets with high affinity and specificity, when folded into their unique three-dimensional structures. They have been first reported more than 20 years ago by Ellington and Gold [1,2]. Ellington reported a selection of RNA molecules that specifically bind to organic dyes, and Gold reported an RNA ligand that interacts with T4 DNA polymerase. Several aptamers have been identified for different targets as diagnostic tools and disease treatment drugs. There is an intense interest and ever increasing need within basic and clinical sciences to detect, analyze and quantify these small molecules and proteins utilizing aptamers. Over 1000 papers related to aptamers have been published each year since 2010, attesting the wide applicability and great potential of aptamers. Figure 1 indicates a large number of scientific publications in this active field of research.

### Aptamer Properties

**Figure 2:**
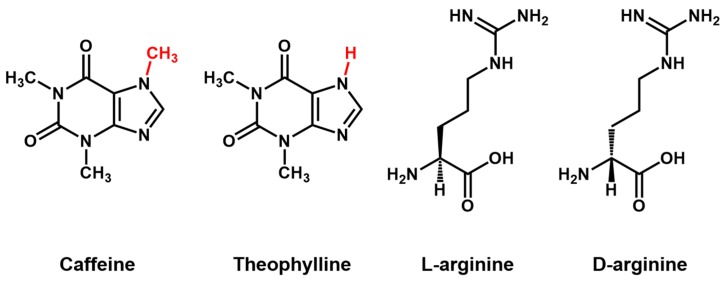
Chemical structure of theophylline, caffeine, *L*-arginine and *D*-arginine.

The word “aptamer” was derived from the Latin *aptus*—meaning “to fit”, and Greek *meros*—meaning “region” [1]. Nucleic acid aptamers are single strands of DNA or RNA (and chemically-modified DNA or RNA) with a length in the range of 10–100 nucleotides (nt), which are identified from an *in vitro* selection process: “systemic evolution of ligands by exponential enrichment (SELEX)” [2]. Over multiple rounds, the SELEX process allows isolation of functional oligonucleotide sequences that can recognize a specific target from a random single-stranded (ss)DNA or RNA library. Within the nucleic acid library, some sequences are folded into unique three-dimensional structures, possessing a combination of loops, stems, hairpins, pseudoknots, bulges, or G-quadruplexes [3,4,5,6]. The aptamer-target recognition was through intermolecular interactions such as aromatic rings, pi-pi system stacking, van der Waals and electrostatic interactions between charged groups and hydrogen bonding. Sometimes it requires the aptamer to undergo adaptive conformational changes and have their three-dimensional structure folded to a unique binding conformation for its target. Aptamers have high target chemical structure specificity, which makes it possible to discriminate a specific molecule from its analogues. Theophylline, a methylxanthine drug, is used for respiratory diseases treatment such as chronic obstructive pulmonary disease (COPD) and asthma. Because of its narrow therapeutic index, serum levels must be monitored carefully to avoid life-threatening toxicity [7]. Theophylline is chemically similar to caffeine, which is present in serum samples. Thus, diagnostic methods must discriminate efficiently among these compounds. The theophylline-binding aptamer shows an affinity for its cognate ligand 10,000-fold higher than that of caffeine, which differs from theophylline by only a single methyl group at nitrogen atom N-7. Enantioselective, a low molecular weight aptamer shows 12,000-fold stronger affinity with *L*-arginine than with *D*-arginine. Figure 2 shows the chemical structures of those small molecules mentioned above [8,9].

Beyond those features, aptamers have shown a number of extraordinary promises as molecular recognition elements in bioanalytical applications. First, due to their thermal stability, aptamers are easy to process and handle. After heat denaturation, the activity and functionality of aptamers can be easily restored within a few minutes since aptamers can undergo reversible denaturation [10,11,12]. Second, unlike amino acid-based receptors such as oligopeptides and antibodies, nucleic acid aptamers can not only be easily amplified by Taq polymerase-dependent polymerase chain reaction (PCR) [13], but also be readily produced by chemical synthesis [14,15,16]. In addition, aptamers can be easily modified by reporter molecules, linkers, and different functional groups on both the phosphate/ribose backbone and the nucleobases to improve resistance to enzymatic degradation, reduce off-target events [17,18,19,20,21,22,23,24,25], and be immobilized on solid-phase substrates or beads for various applications [26,27,28,29]. Moreover, aptamers can also be expressed *in vivo* when the cells contain plasmids that encode an aptamer sequence [30,31,32,33,34]. Lastly, one of the most important advantages of using an aptamer as a probe is that the technique requires no animals or *in vivo* immunization, thus minimizing batch-to-batch variations [35,36].

Aptamer-based biosensors have not only been proven on protein targets for biomedical diagnostic applications, but also been demonstrated with organic and inorganic small molecule compounds, drugs, and antibiotics.

## 2. Generation of Aptamers

The selection of aptamers involves two steps: upstream screening and downstream truncation. The first step is to discover the full-length aptamers from the single-stranded DNA or RNA libraries through the SELEX process. The second is to identify the minimal and essential nucleotides of the full-length aptamers for the target molecule binding.

### 2.1. General Process for Aptamer Screening

**Figure 3:**
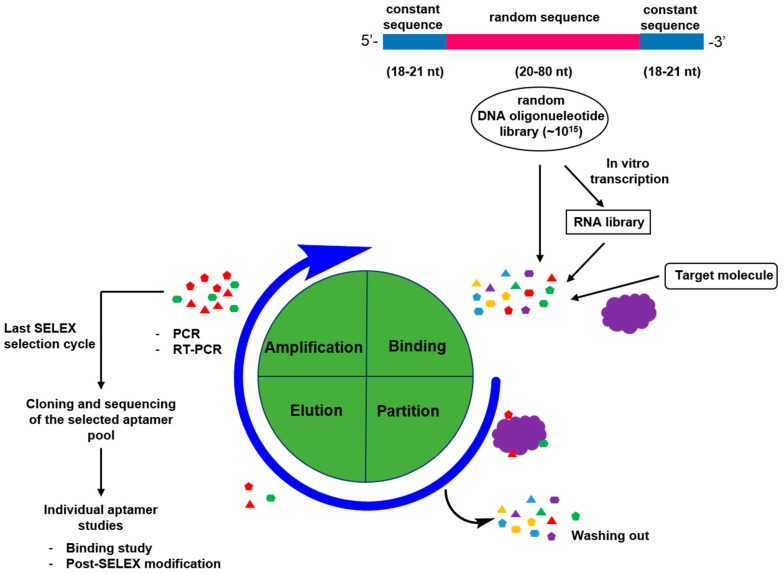
*In vitro* selection of target specific aptamer through SELEX screening process.

Through SELEX process, aptamers have been explored extensively as specific and high affinity probes to a variety of targets, ranging from small organic molecules dyes to large biomolecules such as proteins, cells and even entire tumor tissue [8,9,37,38,39,40,41,42,43,44,45,46,47,48,49,50,51,52,53,54]. The whole process starts from generating a randomized nucleic acid (DNA or RNA) sequence library, which is normally composed of ~10^15^ different aptamers sequences that theoretically can recognize any target molecules [1,2]. Because the efficiency of phosphoramidite chemistry for A, T, G, and C coupling reaction is very similar, the randomized ssDNA library can be generated through a regular DNA synthesizer by using a mixture of phosphoramidites in a ratio of 1.5:1.25:1.15:1.0 (A:C:G:T) [55]. The diversity of the library is determined by the length of random sequence regions at the center flanked by designed primer binding sites at the 5′ and 3′ ends. Even though one can generate 4^n^ different sequences from n nucleotides in principle, about ~10^15^ aptamer combinations can be produced in the library, in practice, corresponding to a random region length of about 25 nucleotides. Figure 3 depicts a typical SELEX process flow, including repetition selection cycle and amplification.

The SELEX procedure consists of binding, partition, elution, and amplification. The starting point of a basic process is synthesizing an oligonucleotide sequences pool. Each sequence in this library has a central randomized sequence (20–80 nucleotides) flanked by fixed primer binding sites (18–21 nucleotides) for PCR amplification. Once the library is created, the library pool is incubated with the target molecule. Some of these oligonucleotides in the library will bind to the target and are then considered aptamers. Unbound nucleic acids are filtered out of the solution, and the bound nucleic acids are separated from the target—This is called elution. Lastly, the binding oligonucleotides are then amplified using PCR to create a new library. Artificial or chemically modified oligonucleotide bases were often used in SELEX experiments to increase the complexity of the library, to introduce new features like functional groups providing new possibilities for the interaction with target molecules, or to enhance the stability by increasing the resistance to nucleases. For example, Wang and co-workers have raised boronic acid modified aptamer for the specific recognition of the glycosylation site(s) of a glycoprotein [56], while Sawai and co-workers adjusted DNA aptamers by using the protonated amino group at the C_5_ position to enhance the binding affinity with the sialyllactose [57]. Generally, in RNA aptamers, the chemical modification of the sugar is realized at the level of the 2′-OH group of the ribose (2′-NH2, 2′-fluro, or 2′-O-methyl) [58,59]. These modifications could significantly improve aptamer stability for *in vivo* application [60,61]. Spiegelmers, chemically synthesized L-ribonucleic acid-based RNA-like aptamers, are highly considered to be resistant to degradation by nucleases and have shown to be stable over 60 h in biological fluid [62,63]. Three spiegelmer candidates are considered as potential drugs and are currently being tested in clinical trials [64]. In the first round of SELEX selection, the oligonucleotide library and target molecules are incubated for binding. (In this step, the ssDNA oligonucleotide library may be *in vitro* transcribed into a RNA library for SELEX selection if RNA aptamer selection is needed.) During the incubation stage, the target molecules interact with the aptamer library either as a free form or a form that is immobilized on a solid support substrate surface. The fixation of target molecules on a solid support surface helps easy separation of bound nucleic acids to target from unbound or weakly bound nucleic acids. However, the immobilization of the target molecules may result in molecular structure or conformation changes and cause interference on the aptamer library and target molecules binding [65]. The aptamer-target complex formation is followed by partitioning from unbound and weakly bound oligonucleotides, which are removed through several washing steps. This is an important step to screen high binding affinity aptamers from the library pool. The elution of the strong bound nucleic acid aptamers from the target molecules might be difficult, especially through the affinity chromatography type method. The applicability of the method might be restricted to isolation of the aptamers possessing extremely high affinity [66,67]. The target bound oligonucleotides are eluted and followed by PCR or RT-PCR amplification. As a result, an enriched selected oligonucleotide pool is generated from the PCR products and this pool will be used for the next SELEX selection cycle. Typically, 6–20 selection cycles are needed to identify and isolate target specific and high affinity aptamers [68,69]. The last SELEX selection cycle ends at the amplification step and the enriched aptamer pool is cloned for further analysis and post-SELEX modifications to improve the functionality.

### 2.2. Cell-SELEX: Aptamers for Cell Membrane Analysis

The cell membrane is complex and contains various molecules, lipids, carbohydrates, and proteins, which play critical roles in cellular behaviors and activities. The composition, structure, and modification of those biomolecules may be different among cell types and between normal and abnormal cells. Discrimination and unveiling of the cell membrane changes and differences are critical for disease diagnosis, treatment and drug design.

**Figure 4:**
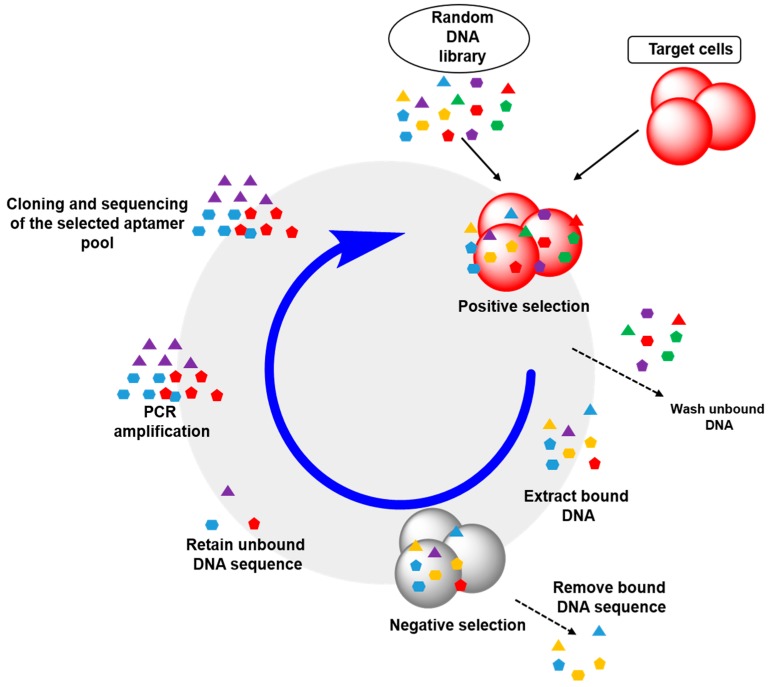
Scheme of cell-SELEX screening process.

Compared to pure target molecules like small molecule organic dyes or proteins, cell membrane is a very complex target system. Cell-SELEX identifies aptamers that specifically bind to a certain cell type based on unique cell membrane extracellular characteristics. As a result, this method requires no cell surface biomarker information prior to the experiment. In 1998, Gold group [39] reported the first experiment using cell membrane as a selection target. They used red blood cell to bind aptamer library and demonstrated that SELEX method could be used not only on pure molecules, but also on complex objects such as whole cells. Recently, cell-SELEX has been further utilized for cancer research, which is particularly useful for identifying subtle cell proteomic changes between normal and cancerous cells. The positive selection on cancer cells, and negative selection on normal cells, can reveal molecular differences among the proteins. Shao group [70] has performed cell-SELEX experiment on paraffin-embedded carcinoma tissue samples. This selection process generated an aptamer, BC 15, which can specifically recognize breast cancer cells which express hnRNP A1 protein. The aptamer works for both MCF-7 cell line and clinical samples. The general aptamer screening procedure of cell-SELEX is shown in Figure 4.

### 2.3. Aptamer Truncation

A full length aptamer selected from nucleic acid libraries is generally ~80 nucleotides long but the region that plays the role of target contacting is usually 10–15 nucleotides [71,72]. In certain conditions, the existence of nonessential nucleotides could interfere with the aptamer and target molecule binding [73]. Thus, the redundant and nonessential nucleotides should be eliminated. Many strategies have been employed for probing and minimizing oligonucleotide sequences length without losing the high binding affinity. Computational algorithms were used to predict the truncated aptamer sequences and secondary structures. For example, Giangrande and co-workers have used a “rational truncation” approach guided by RNA structure prediction and RNA/target docking algorithms to truncate an RNA aptamer to prostate-specific membrane antigen (PSMA), which retains protein binding activity and functionality [74,75,76,77,78,79,80]. Other techniques such as partial fragmentation were used to determine the minimal binding sequence of DNA aptamers for platelet-derived growth factor (PDGF) protein [81]. DNase I footprinting analysis was used to reveal the prion protein (PrP) binding region of selected DNA aptamers [82,83]. Also microarray based quantitative and high-throughput binding sequence determination strategy was employed to select aptamers for immunoglobulin E (IgE) and human cancer biomarker angiopoietin-2 (Ang2) [84,85]. However, the usefulness of those elegant approaches is compromised since they are expensive, complicated for operation, and time-consuming. Recently, the hybridization inhibition approach was developed to identify the minimal binding sequences. Wang and co-workers used the complementary oligonucleotide probes to hybridize with the nonessential region of the anti-human protein tyrosine kinase 7 (hPTK7) aptamer to generate truncated aptamers [73]; Duan and co-workers used the same approach to generate truncated aptamer for CD133 marker of cancer stem cell [86].

## 3. Aptamers as Sensors for Biomarker Discovery

Biomarkers indicate disease pathogenesis progression state, the risk of a particular disease, and the change of gene or protein expression level when physiological conditions change. Biomarkers can have multiple forms, among which proteins and unique genomic DNA sequences are the most popular ones. For example, HER2/neu gene expression level could be used to predict a patient’s response to breast cancer treatment, while the BRCA1 gene mutation could be used to estimate breast cancer development risk [87,88,89]. However, a very limited number of biomarkers have been used clinically and novel biomarker discovery methods are needed to improve their use in clinical stages. Conventional approaches for biomarker discovery use mass spectrometry or monoclonal antibody (mAb), which enables researchers to analyze the cellular proteome or metabolome and identify disease specific molecules or metabolites [35,36,90,91]. However, membrane proteins are still hard to tackle. mAb has been utilized to validate biomarkers by specifically recognizing proteins that are significantly expressed in diseased or abnormal cells. However, mAb requires a lot of information regarding the target molecules. Developing a mAb for a specific cell type with unknown protein epitopes is nearly impossible by the standard antibody development procedure. Compared with mAb, aptamers are able to target cells through the exponential enrichment process, which generates a highly specific DNA sequence by multiple rounds of selection.

The general practice in aptamer-assisted biomarker discovery is based on the following steps. First, the target cells have to be amplified, collected and lysed. The cell components like cell debris or membrane proteins are incubated with biotinylated aptamers and subsequently the biomolecule-aptamer complex is isolated by streptavidin-coated resin or magnetic beads, and the bounded proteins are eluted and characterized by SDS-PAGE. The aptamer binding protein band is excised, enzymatically digested and analyzed by mass spectrometry for protein identification. Lastly, the identified protein target is further confirmed by an existing and known antibody. Using the cell-SELEX screening method, many research groups have found aptamers that specifically bind to different membrane proteins such as tyrosine kinase 7 from T-cell acute lymphoblastic leukemia, tenascin-C from glioblastoma cells, immunoglobulin heavy μ (mu) chain from Burkitt’s lymphoma cells and Stress-Induced Phosphoprotein 1 from ovarian cancer [41,92,93,94]. Although this selection approach has been effectively demonstrated to discover molecules as biomarker candidates, the identified protein targets still need to be further characterized and validated with clinical evidences to prove their viability as biomarkers.

## 4. Aptamers as Molecular Imaging Probes

### 4.1. Aptamer-Based Molecular Beacons

The first molecular beacon was introduced to recognize and report the presence of specific nucleic acid target in a homogeneous solution by Tyagi and co-workers [95]. A single-stranded DNA or RNA probe sequence, carrying complementary bases at the 5′ and 3′ end, was labeled at the terminal positions with a fluorescent moiety and a quencher. A stem-and-loop structure was formed within the molecule and the loop portion of the molecule is a probe sequence that is complementary to the target nucleic acid. On the other hand, the resulting close proximity of the stem led to fluorescence quenching. When the specific complementary target was present and hybridized to the loop, the beacon underwent a spontaneous fluorogenic conformational change and the loop separated the fluorophore and the quencher, generating a fluorescence signal to be detected. Inspired by the molecular beacon approach for DNA sequence detection, Tan and co-workers applied this to study protein-DNA interactions and for protein bioanalysis. The interaction between single-stranded DNA binding protein (SSB) and molecular beacon results in significant restoration of the fluorescence of the molecular beacon [96]. Stanton and co-workers utilized this approach for thrombin detection and termed this strategy as “aptamer beacon”. Similar to the molecular beacon, the aptamer beacon possesses two or more conformations, one of which allows target protein binding. A thrombin aptamer was engineered by adding nucleotides to the 5′-end, which are complementary to nucleotides at the 3′-end of the aptamer, forming a stem-loop structure. In the presence of thrombin, the aptamer beacon formed the target-binding structure and the binding of thrombin induced conformational change of the aptamer, which increased the distance between the fluorophore and the quencher and therefore produced fluorescence signal. This assay might be interfered by nonspecific protein binding (e.g., SSB protein) [97]. This is a standard signal “turn-on” approach, essentially based on target recognition and fluorescence enhancement detection. A fluorescence signal quench “turn-off” approach for real-time thrombin detection was further developed by Tan and co-workers. Significant fluorescent signal change was observed when the thrombin aptamer beacon was bound to target, which was attributed to a significant conformational change in the beacon from a loose random coil to a compact unimolecular quadruplex [98]. To prevent undesirable target-beacon interactions or fluorescence quenching in the aptamer sequence, some prior knowledge of the prediction of secondary or tertiary structures of the aptamer beacon are usually required. To overcome this limitation, Li and Nutiu reported a “structure-switching signaling aptamer”. They designed an aptamer-based fluorescent reporter that functions by switching structures from DNA/DNA duplex to DNA/target complex. The fluorophore was originally quenched when no thrombin was present. When the thrombin is present, there is an aptamer structure switching. Upon binding, the aptamer releases the quencher and the fluorescence signal is restored [99]. This approach was further applied for real-time activation and amplification for fluorescence imaging and targeting therapy. Chu and co-workers developed an activatable theranostic beacon by using a structure-switching aptamer triggered hybridization chain reaction (HCR) on the cell surface. The HCR not only amplifies fluorescence signals from a fluorescence-quenched probe for activatable tumor imaging but also accumulates high-load prodrugs from a drug-labeled probe and induces its uptake and conversion into cisplatin in cells for selective tumor therapy [100]. More aptamer beacons were designed and reported based on the above concept. Aptamer beacon labeled quantum-dot was designed for detection of proteins, cancer cell imaging and therapy, drug and viral DNA sensing [100,101,102,103]. Gold nanoparticle (AuNP)-based colorimetric or Förster resonance energy transfer (FRET) methods have been recently developed for many analytes because of the ease of detection, high sensitivity, and potential for high-throughput analysis [104,105,106,107,108].

### 4.2. Optical Molecular Imaging with Aptamer-Based Probes

Optical molecular imaging, including fluorescence and bioluminescence enables visualization of molecular processes on the cellular or tissue level in small animals. It is a key modality to understand physiological or disease processes and provide invaluable information and insight to disease treatment. Aptamer probes have been designed to be activated by specific stimuli or environmental conditions. When the target is present, the aptamer binds to its target and goes through the conformational change, which allows emission of optical signals.

Like antibody-based molecular probes such as enzyme-linked immunosorbent assay (ELISA) and Immunohistochemistry stain (IHC), aptamers can also be used for biomedical and clinical research [109,110,111,112]. Moreover, aptamers have several additional advantages over antibodies as molecular probes. First, aptamers are much smaller molecules than antibodies. The molecular weight of aptamers are usually between 6 and 30 kDa, much smaller than that of antibodies (~150 kDa) [113,114]. Its smaller size can yield greater tissue penetration ability. Second, aptamers are folded into their unique three-dimensional structures spontaneously, making them more resistant to pH, temperature, or other environmental changes than antibodies. In contrast, once antibodies have been denatured, they usually cannot regain their original structure, thus losing their target recognition and binding ability. In addition, the superior chemical stability of aptamers allows them to be more easily modified through traditional chemical means than antibodies. Finally, unlike antibody probes, aptamers are synthesized chemically without any *in vivo* process, which improves their reproducibility and reliability.

Aptamers have no optical properties by themselves, so they have to be labeled with fluorophores, fluorescent quantum dots, contrast agents like radioisotope-containing compounds, or paramagnetic iron oxide nanoparticles for imaging purpose [115,116,117,118]. Fluorescent dyes can be easily conjugated to either 5′ or 3′ ends of the aptamer molecules by phosphoramidite chemistry. For *in vivo* imaging, fluorescent dye labeled aptamers can circulate in the model animal until binding to the specific cells or tissue. In 1997, aptamers were first demonstrated as imaging probes for *in vivo* study to find the rat inflammation tissue by researchers from NeXstar Pharmaceuticals. The technetium-99m (^99m^Tc)-labeled aptamer was selected and targeted at human neutrophil elastase to image inflammation areas. When compared with a radiolabeled IgG, the aptamers probe provides higher signal-to noise (S/N) ratio [119].

**Figure 5:**
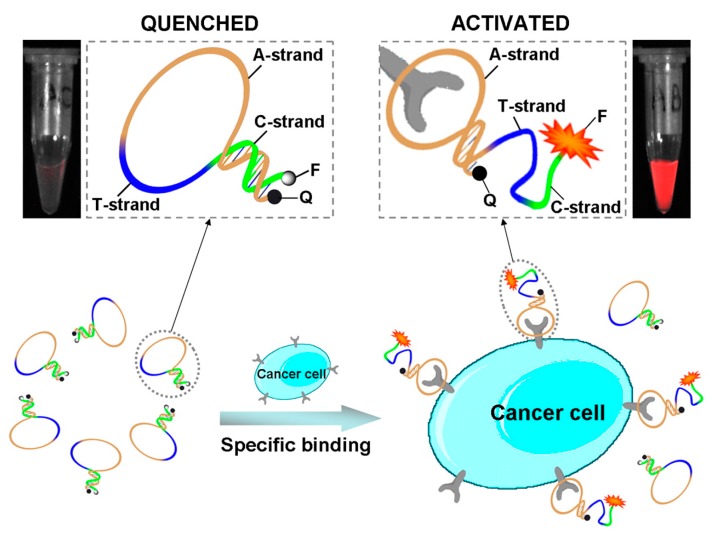
Schematic representation of the design of the activatable aptamers probe (AAP) [124].

The aptamers-based molecular imaging probes have been used to image disease associated biomarkers such as integrins, prostate-specific membrane antigen (PSMA), and nucleolin [120,121,122,123]. Wang group reported an activatable aptamer probe (AAP) with conformation change due to aptamer-protein binding [124]. The researchers engineered an AAP, sg8, which binds to CCRF-CEM lymphoblastic lymphoma cells. The sg8 aptamer is composed of three components: an aptamer sequence that binds to cancer cells (A-strand), a poly-T linker (B-strand), and a short DNA sequence (C-strand) complementary to a part of the A-strand, with a fluorophore and a quencher conjugated at either 5′ or 3′ terminus. In the absence of a target, the aptamer probe is free and its hairpin structure keeps the fluorophore near the quencher, without giving any fluorescence signal. However, once the probe binds to the membrane protein of the target cancer cell, its conformation is changed, leading to an activated fluorescence signal. Figure 5 shows the design and activation of AAP probe.

### 4.3. Aptamer-Based Nanoimaging Agents for CT and MRI

The advance of nanoimaging medicine relies heavily on the development of novel biocompatible organic and inorganic nanomaterials like block copolymers, liposomes, quantum dots (QDs), single-wall carbon nanotubes (SWCNTs), gold nanoparticles (GNPs), and magnetic nanoparticles [115,125,126,127]. All these nanomaterials have unique physical or chemical properties for diagnostic or therapeutic applications. Combining these nanomaterials with aptamers can result in unprecedented characteristics for *in vivo* optical imaging (e.g., with QDs) and photothermal therapy (e.g., with GNPs and SWCNTs).

Aptamer-nanomaterials probes have been used in Computed Tomography (CT) and Magnetic Resonance Imaging (MRI). CT and MRI are the most useful and popular imaging techniques for biomedicine. The Indium-111 (^111^In) labeled aptamer-hollow gold nanosphere (HAuNS) for head and neck cancer detection through CT scan was reported [126]. Epidermal growth factor receptor (EGFR) targeting aptamers were conjugated to HAuNS by attaching a thiol-terminated single-stranded DNA to the HAuNS to generate specific aptamer-HAuNS nanoparticles. In the xenograft tumor-bearing mouse model experiment, ^111^In-labeled aptamer-HAuNS nanoparticles showed greater cancer cell uptake efficiency than ^111^In labeled antibody-HAuNS nanoparticles did. Besides the head and neck cancer cell detection, an A10 aptamer probe and gold nanoparticle conjugate to target PSMA of prostate cancer cell was also reported for CT imaging.

For MRI-based molecular imaging, aptamers have been conjugated to paramagnetic materials such as iron oxide nanoparticles. The A10 aptamer has been labeled on the paramagnetic nanoparticle to target PSMA-expression prostate cancer cell with high sensitivity and specificity. Recently, the Haam group conjugated a vascular endothelial growth factor receptor 2 (VEGFR2)—Specific aptamer on magnetic nanocrystal surface for detection of the angiogenic vasculature of glioblastoma via magnetic resonance imaging. The *in vivo* mouse model test demonstrated aptamer-conjugated magnetic nanocrystal has excellent MRI sensitivity with no cytotoxicity [128].

## 5. Aptamers as Vehicles for Drug Delivery

### 5.1. Aptamer-Polymer Hybrid Delivery System

With great biocompatibility, the use of aptamer and synthetic copolymers are considered as attractive materials for clinical application. Langer group developed an aptamer-polymeric nanoparticle bioconjugate containing a poly (lactic acid)-block-polyethylene glycol copolymer with a terminal carboxylic acid functional group (PLA-PEG-COOH) and encapsulated rhodamine-labeled dextran within the nanoparticle [129]. The bioconjugated nanoparticles can efficiently target and enter prostate LNCaP epithelial cells that overexpress PSMA antigen protein within two hours. Using the same cancer model, they further generated docetaxel (Dtxl)-encapsulated nanoparticles formulated with poly (D, L-lactic-co-glycolic acid)-block-poly (ethylene glycol) (PLGA-*b*-PEG) copolymer and surface functionalized with the A10 aptamers that recognize the PSMA protein as well [121]. This Dtxl-encapsulated aptamer-nanoparticle showed significantly enhanced *in vitro* cellular toxicity for prostate cancer cells. Moreover, a single intratumoral injection of aptamer-nanoparticle bioconjugates resulted in a drastically reduced tumor burden. Five of seven mice treated with such bioconjugates showed complete tumor regression, whereas only two of seven mice showed regression in the group of non-aptamer targeted bioconjugates.

A similar aptamer-PLGA bioconjugate nanoparticle system was used to deliver paclitaxel for glioma treatment [130]. A DNA aptamer, AS1411, which specifically binds to nucleolin that was highly expressed in the plasma membrane of both cancer cells and endothelial cells in angiogenic blood vessels, was conjugated to the surface of PEG-PLGA nanoparticles. The bioconjugates showed significantly higher efficacy compared with the conventional paclitaxel drug *in vivo*, evidenced by reduced tumor size and increased animal survival rate. In summary, the aptamer-polymer conjugates have shown promise in clinical applications for drug delivery, as both PEG and PLGA have been well studied in many US Food and Drug Administration (FDA) approved formulations [131,132,133].

### 5.2. Aptamer-Liposome Hybrid Delivery System

Liposomes are significant pharmaceutical carriers for drug delivery. Being the most successful drug-delivery systems over the past thirty years, many liposome-based drugs have been approved by the US FDA to treat diseases and many more have been in clinical trials [134,135,136]. As aptamer-liposome bioconjugates, cholesterol-modified aptamers have become commercially available, attributed to the attractive property that aptamers can be spontaneously anchored on the outer shell during the conjugated nanoparticle formation [137]. This advantage has been utilized to engineer liposomes to deliver cisplatin and taxol. Cisplatin is a great chemotherapeutic agent to treat a broad range of tumors. Despite its excellent antitumor efficacy, cisplatin has major drawbacks of lacking cell specificity and severe adverse effects. The AS1411 aptamer-liposome bioconjugate system can effectively deliver cisplatin to specific cell targets to mitigate these negative effects. Using aptamer-liposome bioconjugates as carriers for cisplatin, the selectivity of cisplatin was greatly enhanced, as evidenced by effective killing of the target cancer cells but not the control cells [138]. Compared to non-targeting liposomes, the AS1411 aptamer-functionalized liposomes that contain taxol have increased cellular uptake rate and cytotoxicity for MCF-7 breast cancer cells. Athymic nude mice bearing xenograft MCF-7 tumors treated with intratumorally injected aptamer-functionalized liposomes exhibited an earlier onset of tumor inhibition and improved anticancer efficacy [139].

A bone tissue specific aptamer-functionalized liposome nanoparticle system for therapeutic siRNA delivery has been developed. Metabolic skeletal disorders associated with impaired bone formation presents a clinical challenge. RNA interference-based approaches aimed at promoting osteoblastic bone formation holds promise for a cure of this disease. Plekho1 protein has been identified as an intracellular negative regulator of bone formation and Plekho1 siRNA was shown to promote bone formation. An osteoblast-specific aptamer CH6 was selected by cell-SELEX and conjugated to liposome nanoparticles encapsulating osteogenic Plekho1 siRNA. This aptamer-liposome can bind with osteoblast and specifically deliver siRNAs to facilitate bone formation for both osteopenic and healthy rodents [140].

### 5.3. Aptamer-Dendrimer Hybrid Delivery System

Dendrimers are a unique type of synthetic globular macromolecules, which consist of three parts from the interior to the surface: a central core, a repeated and hyperbranched mantle, and a high density of surface functionalities [141,142,143]. Unlike conventional polymer, they possess several favorable features for use in drug delivery, including well-defined molecular weight, confined nanoscale size, multivalency, monodispersity, ease of preparation and modification, high aqueous solubility and high drug-loading capacity [144,145,146,147]. Recently, the combination of conventional chemotherapy with immunotherapy (chemoimmunotherapy) has proven a novel and effective cancer treatment strategy, providing invaluable preclinical and clinical outcomes [148,149,150]. Jon and co-workers reported an aptamer-dendrimer bioconjugate based nano-delivery system for effective chemoimmunotherapy. A G4 polyamidoamine (PAMAM) as carrier, an RNA aptamer that specifically recognizes PSMA was used as a prostate cancer targeting moiety, Doxorubicin (Dox) was chosen as chemotherapeutic agent, and the unmethylated CpG duplex oligonucleotides (dONT) as an immuno-stimulant. The unique resulting aptamer-dendrimer bioconjugate allowed Dox to be loaded via an intercalating interaction between double stranded CG base pairs and was specially delivered to PSMA-overexpressed prostate cancer. Serum stability is also an essential prerequisite for drug-delivery vehicle development. Jon and co-workers also indicated enhanced stability of RNA aptamer in serum due to attachment to dendrimer. This is attributed to the steric hindrance generated around aptamer-dONTs attached to the dendrimer core, which prevented nucleases access to the cleavage site of the bioconjugate [147].

Besides the above mentioned studies, several aptamer-based bioconjugate systems including gold-nanostars [151], single-walled carbon nanotubes (CNT) [152,153], gold nanoparticle-hybridized graphene oxide (AuNP-GO) [154], superparamagnetic iron oxide nanoparticles (SPION) [155], quantum dots [102], and gold nanoparticles (GNPs) [156] for drug delivery, imaging or therapy have been reported. Although the results of studies have been promising so far, more *in vivo* evaluation and validation experiments are still required.

## 6. Aptamers as Potential Drugs

### 6.1. Therapeutic Aptamers in Cancer Therapy

In the clinic, aptamer-based therapeutics is gaining momentum, which can be used as conventional therapeutic drugs in the same way as monoclonal antibody. Macugen (pegaptanib), a selective vascular endothelial growth factor (VEGF) antagonist, was approved in 2004 by the US FDA for the treatment of neovascular (wet) age-related macular degeneration (AMD) [157,158,159]. Today, more candidates were in the pipeline undergoing clinical trials for disease treatment, ranging from cancer to infectious pathogens. Bruno [160], Byrne and Wower [161], and Zu [162], have recently summarized the therapeutic aptamers which are in the clinical trials or in pharmaceutical development pipelines. The following section focuses on the potential therapeutic aptamers in the cancer immunotherapy field.

Programmed cell death-1 (PD-1; also known as CD279) [163,164] and cytotoxic T-lymphocyte-associated protein-4 (CTLA-4; also known as CD 152) [165,166,167] are negative regulators of T-cell activity and limit the activity of T-cells through interaction with their ligands. They act as brakes in the immune system, allowing cancer cells to escape it [168,169,170]. Blocking the immunoinhibitory pathways using monoclonal antibodies has led to provoke robust and durable antitumor responses. Anti-CTLA-4 (Ipilimumab; Yervoy) [171,172] and Anti-PD-1 (Nivolumab; Option and Pembrolizumab; Keytruda) monoclonal antibodies have been approved by the US FDA for melanoma (a type of skin cancer) treatment [172,173,174] and are been currently tested in various tumor types [175,176]. Since monoclonal antibody therapy inherently carries a number of disadvantages including the limitation of using protein-based biologicals might be restricted by their potential immunogenicity, which could elicit neutralizing antibodies in patients. In addition, antibodies are cell-based products, which require complex and costly manufacturing technologies [177,178,179,180,181]. Alternative aptamer-based immunomodulators have been isolated and tested. The first aptamer, Del 60, was developed to bind and block murine CTLA-4 on activated T cells [182]. The monovalent aptamer bound to its target in solution has an approximate K_d_ of 33–60 nM that is comparable with the bivalent antibody (K_d_ ~ 133 nM). Therefore, Del 60 is able to reverse CTLA-4-mediated inhibition of T-cell proliferation *in vitro*. The *in vivo* bioactivity of Del 60 aptamer can be significantly enhanced by generating the tetravalent derivative. Most important, the tetravalent Del 60 aptamer potentiates protective immunity in mice tumor model with comparable potency to CTLA-4 monoclonal antibody. The murine PD-1 aptamer, MP7, was also developed. This DNA aptamer was specifically bound to murine extracellular domain of PD-1 and interrupted the PD-1/PD-L1 interaction. The 5′ end of MP7 aptamer was modified by high molecular weight polyethylene glycol (PEG) to reduce the filtration rate and extended half-life, and without losing the ability to block PD-1/PD-L1 interaction. In addition, the PEGylated MP7 retained the ability to suppress the growth of PD-L1 (+) colon tumor cells *in vivo* with the comparable potency to anti-PD-1 antibody. Notably, the PEGylated MP7 was not cytotoxic and could enhance tumor-specific T-cell response without induction of Toll-like receptor 9 (TLR-9)-mediated innate immune response [183]. An aptamer to the T-cell major costimulatory receptor, 4-1 BB, was also isolated. This protein was repressed on the activated CD8 (+) T-cells [184,185,186,187]. The agonistic 4-1 BB aptamer that functions as the natural ligand to initiate a positive signal transduction cascade to enhance the survival of the CD8 (+) T-cells and induce tumor regression [188].

### 6.2. Thrombin Binding Aptamers

Thrombin, a multifunctional serine protease, is a significant target for anticoagulation and cardiovascular diseases therapy [189]. It plays a key role in the coagulation cascade reaction, converting soluble fibrinogen into insoluble fibrin proteins [190] and also involving many other coagulation catalyzing reactions, including platelet activation [191,192,193]. Thrombin recognizes substrate via two electropositive surfaces (fibrinogen-binding site, exosite I; heparin-binding, exosite II) composed essentially of basic amino acid residues, which are located on the opposite side of the active site of thrombin [194,195]. The inhibition and regulation of thrombin activity *in vivo* are the major solution in prevention and treatment of blood clotting abnormalities. The first DNA thrombin aptamer was isolated by Toole and co-workers in 1992, named thrombin binding aptamer (TBA) and the most active strand was a 15-mer oligonucleotide with a K_d_ ~ 100 nM [113]. The structure of the TBA was determined to be a intramolecular G-quadruplex, which interacts with the fibrinogen-binding site [196,197,198,199,200]. More new aptamers with higher affinity were isolated that contained a duplex region in addition to the quadruplex [201]. Nucleotide analogues such as non-natural isoguanosine (isoG), 5-hydroxymethyl-2′-deoxyuridine (hmU) [202,203], Dansyl/Cyclodextrin moieties [204], unlocked nucleic acid (UNA) [205] were also introduced to enhance the TBA affinity and biological activity. The first *in vivo* TBA anticoagulant properties were evaluated on cynomolgus monkeys and sheep [206]. The rapid onset of action and short half-life (t_1/2_ ~ 2 min) *in vivo* suggest that the thrombin aptamer may be useful in anticoagulation with extracorporeal circuits and acute clinical settings like surgical interventions. It also showed the ability to inhibit clot-bound thrombin activity and platelet thrombus formation in an *ex vivo* whole artery angioplasty model. It also represented specific dose-dependent inhibition of thrombin-induced platelet aggregation ability in human platelet-rich plasma [207]. Currently, the pre-clinical study is being evaluated by Archemix Corporation in preparation for human clinical trials [208]. Nu172 is another potentially thrombin inhibitor candidate, which was isolated from SELEX process and subsequently truncated to 26 nucleic acids without further chemical modification. It is currently being prepared for evaluation in phase II clinical trials for anticoagulation in patients undergoing coronary artery bypass graft (CABG) surgery treatments by ARCA Biopharma [209].

## 7. Aptamer-Based Programmable Hydrogels

The clinical pharmacokinetic (PK) and pharmacodynamic (PD) efficacy of therapeutics is affected by many factors, including drug concentration at the site of action, pathological area regional pH, therapeutics solubility and stability, cellular uptake efficiency, cell membrane permeability, bioavailability, and renal clearance rate [210,211]. In recent years, there has been an increasing effort in the development of environmental-responsive bionanomaterials to facilitate targeted drug delivery and release control, including pH [212], redox reaction [213], enzyme [214,215], small molecule [216,217], temperature [218,219], and light [220,221]. Many review articles summarized progress in this research field [222,223,224,225]. Hydrogel, which can be synthesized from a variety of biological based materials, including polypeptides [226,227], polysaccharides [228,229], oligonucleotides [230,231], is one of the most attractive materials. However, since most hydrogel is highly permeable, it causes rapid release of loaded therapeutics in the tissue [232,233]. Current solution mostly depends on the control of pore size or disassembly rate of the material. However, it is still challenging to utilize those mechanisms to release drugs with temporal and spatial control and specificity. Aptamer-functionalized hydrogels can be programmed to release various and multiple therapeutics when needed through specific nucleic acid recognition and complementary hybridization process. Wang and co-workers recently developed a nucleic acid-based affinity hydrogel system for controlled protein release. Two different nucleic acid aptamers and complementary sequences were used to control the release rate of vascular endothelial growth factor (VEGF) and platelet-derive growth factor BB (BB) [234,235,236,237] *in vitro*.

The aptamer-functionalized hydrogel has also been used for controlled cell capture and release. The programmable hydrogel contains a primary complementary sequence (CS), an aptamer sequence, and a secondary CS. Initially, the primary CS was conjugate to the hydrogel on a solid support surface. The aptamer was hybridized with the primary CS and can specifically bind with cells through polyvalent aptamer-protein interactions. When the secondary CS was applied to trigger the hydrogel, the aptamer dissociated from the primary CS and hybridizes with the secondary CS. Therefore, polyvalent interactions between the cells and the hydrogel were weakened, causing nondestructive detachment of the cells from the solid surface [237]. In addition, aptamer-functionalized hydrogel has also been used as an artificial extracellular matrix (ECM) for cell adhesion without affecting cell viability [238].

## 8. Aptamers in Precision Cancer Medicine

Conventional approaches of clinical practice are drug centered, with a strategy of finding generalities between patients so that they can be clustered or grouped together and treated similarly [239]. Precision medicine is a new approach to integrate multiparameters including molecular, clinical, environmental data, and health outcomes to enhance disease diagnosis and treatment in an iterative fashion [240,241]. The potential of precision medicine is that it will yield treatments that deliver significant effects based on molecular characteristics of patients, rather than just the organ of origin. Oncology is the frontier of precision medicine with significant emphasis on identification of tumor-associated biomarkers [242,243,244,245,246]. Proteins are the most useful forms of biomarkers giving information about the genotype and phenotype of a certain disease. The majority of protein-based biomarkers are found in blood and body fluids, and the discovery of such biomarkers is crucial in providing physicians with essential information to diagnose diseases and treat patients. However, a very limited number of validated biomarkers have been utilized in routine practice, such as human epidermal growth factor receptor 2 (HER2/neu) in breast cancer [247], prostate-specific antigen (PSA) in prostate cancer [248], carbohydrate antigen 125 (CA-125) in ovarian cancer [249], and K-Ras mutation in colorectal cancer or non-small-cell lung cancer (NSCLC) [250,251]. Moreover, biomarkers are often found at low-abundance, and therefore the detection method should be sensitive enough to distinguish between protein isoforms and molecular structure difference through post-translational modification [252,253]. If cancers are defined or classified by their molecular makeup, advanced molecular tests should be considered standard diagnostic tools for patients with cancer [239]. In addition, new technologies for rapid biomarker detection are needed. An enzyme linked oligonucleotide assay (ELONA) was first reported by Drolet and co-workers using aptamer-based sandwich assay to replace antibodies to detect human vascular endothelial growth factor (hVEGF) protein [254]. Recently, several groups have reported aptamer-based tumor marker discovery platforms with versatile development potential or multiplexing capabilities. Yang and co-workers have developed an aptamer-functionalized graphene oxide to detect platelet-derived growth factors (PDGF) [255]. Walker and co-workers have recently described a slow off-rate modified aptamer (SOMAmer) biomarker discovery platform that is capable of simultaneously measuring thousands of proteins from serum or plasma with small sample volume. By using this system, 58 potential chronic kidney disease (CKD) biomarkers were discovered [256]. They also utilized this platform to conduct a large scale study of NSCLC biomarkers screening from which 44 biomarkers were discriminated [257]. Aptamer-facilitated biomarker discovery (AptaBiD) technology was introduced to detect biomarkers differently-expressed from immature and mature dendritic cell membrane. Through multi-cycle selection, the biomarkers were isolated and subsequently analyzed by mass spectrometry. Tan and co-workers summarized the development of nucleic acid aptamers in the areas of cell membrane analysis, cell detection and isolation for biomarkers discovery [258]. Liu and co-workers summarized the recent research progress of aptamer-based biosensors for biomedical diagnostics [259] and Tang and co-workers reviewed the development of aptamer-based enzyme-linked immunosorbent assay (ELISA) for biomolecules detection field [260].

Novel technologies using aptamers continue to evolve in precision medicine, providing enormous opportunities for tumor related biomarker discovery and detection.

## 9. Future Perspective and Conclusions

SELEX and cell-SELEX are effective and versatile techniques to generate a large number of aptamer-based probes that can recognize specific molecular or cell targets even with limited or no information about the markers the aptamers will bind to. These aptamers contribute to biomedical research and practice in the areas of imaging, biomarker discovery, drug delivery, *etc*. Since the fundamental patent for SELEX has recently expired, an increasing number of aptamers and related applications are expected to be developed and more aptamer-based disease diagnostic and therapeutic molecules will be put in the pipeline for clinical development.

Although aptamer-based drug targeting and delivery systems have been proven highly specific to cancer cells, the following questions still need to be answered: (1) how aptamers of high affinity to the disease-associated targets can be more efficiently and rapidly selected; (2) how aptamers can be engineered to maintain their correct conformation and structure on the surface of nanoparticles or liposomes; (3) how the shape or size of the nanoparticles can affect the target binding ability of aptamers; and (4) how *in vivo* biostability of aptamers can be improved to be suitable for clinical use.

In summary, aptamers are highly attractive and promising tools for functional characterization of biomolecules, disease detection, therapeutic intervention as drug carriers, and the pharmaceutical lead compounds. Aptamers have significant translational potential to be an essential part of safe, controllable, and robust drug delivery systems. In many fields of biomedical research, aptamers have been playing key roles and will continue to open up new possibilities unconceivable today.
